# Taurine-mediated metabolic immune crosstalk indicates and promotes immunosuppression with anti-PD-1 resistance in bladder cancer

**DOI:** 10.3389/fimmu.2025.1618439

**Published:** 2025-06-24

**Authors:** Zhengfang Liang, Fengwei Nong, Zhenjie Li, Runmin Chen, Haoxu Zhao, Yongping Huang

**Affiliations:** ^1^ Department of Urinary Surgery, Affiliated Hospital of Youjiang Medical University for Nationalities, Baise, Guangxi, China; ^2^ The First Affiliated Hospital of Jinan University, Jinan University, Guangzhou, Guangdong, China; ^3^ Department of Renal Diseases, Affiliated Hospital of Youjiang Medical University for Nationalites, Baise, Guangxi, China; ^4^ Key Laboratory of Tumor Molecular Pathology of Baise, Affiliated Hospital of Youjiang Medical University for Nationalities, Baise, Guangxi, China

**Keywords:** bladder cancer, spatial transcriptomics, taurine, CAFs, anti-PD-1

## Abstract

**Background:**

Bladder cancer (BLCA) remains heavily dependent on bacillus Calmette-Guérin (BCG) therapy due to the profound heterogeneity of its tumor microenvironment (TME) and deregulated metabolic landscapes. Taurine metabolism (TM) is a pivotal axis in BLCA, exhibiting dual roles in tumor progression and immune evasion. Deciphering the molecular mechanisms by which TM reprogramming fosters immunosuppression is imperative for advancing BLCA immunotherapy.

**Methods:**

This study employed an integrative approach combining single-cell RNA sequencing (scRNA-seq), spatial transcriptomics (ST), and bulk transcriptome analyses to unravel taurine metabolic dysregulation in the BLCA TME. Computational frameworks such as Seurat and Monocle 3 were used to characterize cellular subpopulations, reconstruct differentiation trajectories, and model intercellular signaling networks. A taurine metabolic dysregulation index (TMs) was developed using TCGA cohorts, with survival modeling and machine learning methodologies deployed to assess its prognostic utility. Immuno-infiltration patterns and immunotherapeutic responsiveness were quantified via algorithms including ESTIMATE and TIDE. Mechanistic validation was achieved through co-culture systems.

**Results:**

ScRNA-seq profiling revealed significant perturbations in TM scores across epithelial cells, fibroblasts, and macrophages within the BLCA TME. High TMs clusters were enriched for Notch signaling and EGFR tyrosine kinase inhibitor resistance pathways. Spatial transcriptomics analyses highlighted spatiotemporal heterogeneity in taurine metabolic gene expression. The TMs index emerged as an independent prognostic biomarker, with high TMs patients demonstrating significantly shorter overall survival and synergistic prognostic deterioration in the context of high tumor mutational burden (TMB). High TMs tumors exhibited enrichment of immunosuppressive cell compartments and elevated immune checkpoint molecule expression. Mechanistically, FAAH knockdown in cancer-associated fibroblasts (CAFs) attenuated co-cultured BLCA cell viability, potentially mediated by CCL15 secretion.

**Conclusion:**

This study establishes that taurine metabolic dysregulation reconfigures intercellular signaling within the BLCA TME, driving immunosuppression and tumor progression. The TMs framework enables robust patient stratification and provides a mechanistic rationale for therapeutic strategies targeting TM in conjunction with immune checkpoint inhibitors, thus paving the way for advanced precision medicine approaches in BLCA.

## 1 Introduction

Immunotherapy, as a revolutionary force in the field of cancer treatment, has brought new hope to numerous cancer patients. In particular, immune checkpoint inhibitors (ICIs) unlock the immune system’s suppression of tumor cells and activate the killing activity of T cells against tumors by blocking immune checkpoint signaling pathways such as PD-1/PD-L1 and CTLA-4 (1, 2). Despite this promise, clinical outcomes with ICIs remain suboptimal, with only 20–30% of solid tumor patients deriving benefit, while the majority experience treatment failure (3). A defining driver of ICI refractoriness is the immunosuppressive tumor microenvironment (TME), a complex ecosystem comprising tumor cells, immune infiltrates, stromal cells, and extracellular matrix (ECM) that dictates tumor progression and immune evasion (4). The TME of different tumors exhibits high specificity, fostering robust immune resistance. For example, in the TME of triple-negative breast cancer—where endocrine therapy and anti-HER2 targeted therapy show poor efficacy—myeloid-derived suppressor cells (MDSCs) accumulate in large numbers, promoting tumor immune escape by inhibiting the functions of T cells and NK cells. Bladder cancer faces similar challenges (5). Bladder cancer, the ninth most prevalent malignancy globally, exhibits a striking male predominance (3–4-fold higher incidence than females) and strong associations with smoking and occupational exposures. Current therapeutic modalities—including transurethral resection, radical cystectomy, cisplatin-based chemotherapy (e.g., GC regimen), and bacillus Calmette-Guérin (BCG) instillation—yield a dismal 5-year survival rate below 50% for muscle-invasive disease (MIBC), with 40% of patients experiencing recurrence within 3–4 years (3, 6). This clinical inertia stems from the TME’s profound heterogeneity, encompassing tumor cells, cancer-associated fibroblasts (CAFs), endothelial cells, immunosuppressive leukocytes (Tregs, M2 macrophages), and cancer stem-like cells (BCSCs). The ECM, enriched in collagen, fibronectin, and matrix metalloproteinases (MMPs), further accelerates invasion and metastasis (7).Metabolic reprogramming in the TME is characterized by heightened glutamine catabolism, fatty acid anabolism, and dysregulated taurine metabolism, while NF-κB-driven inflammation fuels tumor progression via secretion of proangiogenic cytokines like IL-6 and IL-8 (8).

The multifaceted immune resistance arising from TME cellularity, cytokine signaling, metabolic aberrations, and checkpoint expression underscores the urgent need for mechanistic insights to overcome ICI limitations (5, 9).Advanced technologies have emerged as critical allies in this endeavor: single-cell RNA sequencing (scRNA-seq) has unveiled discrete CAF subsets, such as PDGFRα+ITGA11+ cells that promote lymphatic metastasis via ITGA11-SELE crosstalk, and SLC14A1+ CAFs that enhance chemoresistance through WNT5A secretion (10) Spatial transcriptomics, meanwhile, has illuminated the spatial organization of TME immunity, revealing TLS-enriched CXCL13+ T cells and plasma cells that augment C1QC+ macrophage phagocytosis via IGHG secretion. Notably, tumor cell–restricted taurine transporter SLC6A6 expression correlates with spatial exclusion of CD8+ T cells, implicating metabolic competition as a driver of immune privilege (11, 12).Multi-omics approaches integrating single-cell and proteomic data have further identified semi-squamous differentiation as a marker of MIBC chemoresistance, with cathepsin CTSH emerging as a target for inducing terminal differentiation in resistant clones.

The microenvironmental characteristics of bladder cancer may be investigated by anchoring taurine metabolic disorders, which play a critical role in inflammation-driven tumorigenesis (13). As a sulfur-containing amino acid, taurine exerts dual roles in the tumor microenvironment (TME) of bladder cancer. Under normal physiological conditions, taurine participates in bile acid synthesis within the digestive system. Bile acids are synthesized from cholesterol in the liver, and taurine conjugates with bile acids to form conjugated bile acids, facilitating fat emulsification and absorption (14).In the immune system, taurine enhances macrophage phagocytosis, elevates neutrophil bactericidal activity, and promotes the proliferation and differentiation of T and B cells, thereby strengthening the body’s immune defense capabilities (14, 15).Concurrently, taurine regulates inflammatory responses in immune cells, inhibits damage caused by excessive inflammation, and maintains immune homeostasis. However, in the context of tumor metabolic reprogramming and core oncogenic mutations, taurine assumes unique roles in the tumor microenvironment (16).Tumor cells overexpress SLC6A6 to uptake taurine, leading to reduced taurine levels in CD8+ T cells. This triggers endoplasmic reticulum stress and ATF4-mediated upregulation of PD-1 and TIM3, ultimately inducing T cell exhaustion. Taurine supplementation can reverse this process and synergize with PD-1 inhibitors (15, 17). In terms of inflammation regulation, taurine inhibits the NF-κB pathway to reduce the secretion of pro-inflammatory cytokines such as IL-6 and IL-8, thereby suppressing tumor cell proliferation and angiogenesis. It also regulates macrophage polarization, promoting the transition from M1-type (pro-inflammatory) to M2-type (immunosuppressive) macrophages and reshaping the immune microenvironment (17). Metabolically, taurine deficiency enhances glutamine catabolism, leading to α-KG accumulation and activation of the HIF-1α pathway, which promotes tumor cell glycolysis and invasion. Meanwhile, taurine enhances tumor cell chemoresistance by maintaining mitochondrial membrane potential and inhibiting reactive oxygen species (ROS) production (7, 18).

To this end, this study will integrate scRNA-seq, spatial transcriptomics, and bulk transcriptomic data to systematically dissect the molecular underpinnings of taurine metabolic dysregulation within the tumor microenvironment (TME) of bladder cancer. It aims to address the fundamental question of how aberrant taurine metabolism promotes tumor progression through modulating immune cell function and inflammatory signaling pathways. Technically, the study leverages multidimensional transcriptomic approaches to delineate the spatial expression patterns of taurine metabolism–related genes and their interaction networks with immune cells. Ultimately, this work aspires to achieve clinical translation by developing taurine metabolism–based prognostic biomarkers and exploring therapeutic strategies combining taurine supplementation with immune checkpoint inhibitors, thereby offering new avenues for BLCA treatment.

## 2 Methods

### 2.1 Single-cell data processing, cell subset identification, and annotation

High-throughput sequencing data and clinical information for bladder cancer (BLCA) were obtained from the UCSC Xena platform (https://xenabrowser.net/) (19).The paired single-cell datasets, including normal bladder tissues and cancer tissues from GSE222315, were acquired via the GEO database. To ensure data quality and standardization, batch effect correction and integration of multi-group gene expression data were performed using R packages such as “limma” and “sva”. For single-cell data, the Harmony integration algorithm was applied to eliminate potential experimental batch variations. Additionally, a taurine metabolism-related gene set was systematically screened from the GeneCard database, serving as the foundation for subsequent gene scoring and functional enrichment analyses.

### 2.2 Single-cell data analysis and quantification of taurine metabolic dysregulation scores

Preprocessing of single-cell transcriptome data was conducted using the “Seurat” R package. Low-quality cells were first detected via the “PercentageFeatureSet” function, with single-cell gene expression set to a range of 200–10,000 and mitochondrial gene expression ratio below 20%. Data normalization was then performed using the “NormalizeData” function, converting processed data into Seurat objects for subsequent analysis. Highly variable genes were identified via the built-in “FindVariableFeatures” function, followed by scaling and principal component analysis (PCA) using the “RunPCA” tool. To reveal latent structural features, dimensionality reduction and visualization were further performed using UMAP and t-SNE algorithms. In cell subset analysis, differential expression analysis of distinct cell types was conducted via “FindAllMarkers” combined with Wilcoxon rank-sum tests and manual annotation based on data source annotations, with cell subset annotation completed using feature genes. For assessing purine metabolism characteristics, the aforementioned taurine metabolism-related gene set was integrated with five classic analytical methods—AddModuleScore, single-sample gene set enrichment analysis (ssGSEA), AUCell, UCell, and singscore—to score the endogenous tumor microenvironment of BLCA, with metabolic activity characteristics of each cell subset ultimately visualized via interactive bubble plots.

### 2.3 Taurine-related cell communication and pseudotime analysis in the BLCA microenvironment

Pseudotime trajectory analysis of tumor cells was performed using the “Monocle 3” R package based on dynamic changes in single-cell gene expression data. Specifically, dimensionality reduction was first conducted via the DDRTree algorithm, followed by pseudotime analysis to reveal continuous changes in epithelial cells with high/low taurine expression from initial to terminal states. The “PLOT_CELL_TRACTURE” function was used to dynamically order distinct cell subsets along the pseudotime axis. For intercellular communication analysis, the “CellChat” software was employed to construct ligand-receptor regulatory networks across cell types. Integrating interaction data from CellPhoneDB and STRING databases via the netVisualDiffInteraction algorithm with cell-specific expression profile data, bidirectional regulatory mechanisms between high/low taurine-expressing epithelial cells and immune/stromal cells were revealed, providing a theoretical basis for targeted tumor microenvironment therapy.

### 2.4 Processing of BLCA spatial sections and spatial cell subset annotation

The spatial transcriptome dataset GSE171351 from the GEO database was selected as an independent validation cohort. These datasets included fresh-frozen tumor tissue sections from which high-resolution spatial gene expression matrices were obtained via the 10x Genomics Visium platform. During data preprocessing, normalization and correction were performed using the “SCTransform” algorithm in “Seurat”, followed by dimensionality reduction of standardized UMI count matrices via the “RunPCA” function to identify spatial transcriptomic subsets with similar gene expression patterns. For spatial location information in spatial transcriptome data, deep mining was performed using “stLearn”, a tool developed based on Scanpy. This method integrated cell type annotation, gene co-expression networks, and spatial coordinate information to identify significantly enriched gene modules via spatial autocorrelation analysis. Cell type deconvolution of spatial transcriptome data was performed using a Bayesian hierarchical model based on single-cell reference atlases, effectively distinguishing spatial distribution characteristics of epithelial cells, immune cells, and stromal cells with high/low taurine metabolic features and revealing interaction patterns among cell types in the tumor microenvironment.

### 2.5 Establishment of taurine metabolism population criteria and clinical characteristic evaluation

Based on the TCGA-BLCA cohort, the dataset was stratified and randomly sampled at a 7:3 ratio into training and validation sets. Single-factor Cox proportional hazards regression was first performed via survival analysis R packages to systematically screen genes significantly associated with overall survival (OS, p<0.05). To optimize model complexity, Lasso regression was used for feature dimensionality reduction, with the optimal λ value selected via 1000-fold cross-validation to further screen model-building genes. Combined with multi-factor stepwise regression analysis, key oncogenes were identified to construct a multivariate Cox risk model. Based on this model, a taurine metabolic score (TM score) was calculated for each patient. Survival analysis was performed via the Kaplan-Meier method, with intergroup differences evaluated via log-rank tests. Model predictive efficacy was assessed via time-dependent ROC curves. The final prognostic nomogram integrated three independent variables: TM score, age, and clinical stage. Model stability was validated via Bootstrap resampling, with calibration curves showing high consistency between predicted and observed survival rates.

### 2.6 Immune characteristics of BLCA patients stratified by TM score

The ESTIMATE algorithm was used to calculate stromal score, immune score, and tumor purity for each patient based on expression matrices of 141 stromal and 225 immune signature genes, quantifying tumor microenvironment characteristics by inferring proportions of tumor mesenchyme and immune cells from gene expression data. To systematically evaluate immune infiltration characteristics of BLCA patients stratified by TM score, analysis results from seven mainstream algorithms—including CIBERSORT, MCPcounter, and XCELL—were obtained from the TIMER 2.0 database (20).Activation states of 28 immune cell types were further quantified via single-sample gene set enrichment analysis (ssGSEA). For immune escape assessment, the TIDE computational framework (http://tide.dfci.harvard.edu/) was used to analyze immunotherapy response potential in tumor samples and evaluate immune checkpoint inhibitor efficacy. Multi-dimensional charts were generated using ggplot2 throughout the study.

### 2.7 Construction and validation of bladder cancer co-culture models

Bladder cancer cell line T24 and human bladder tumor-associated fibroblasts hCAF-BT (both purchased from the Chinese Academy of Sciences Cell Bank) were used in this experimental section. T24 cells were cultured in high-glucose DMEM medium with 10% FBS, while hCAF-BT cells were maintained in F12K medium supplemented with 1% PS double-antibiotics. Cells were cultured in a 37°C, 5% CO_2_ incubator and passaged every 2–3 days. For Transwell co-culture system construction, log-phase T24 cells were seeded in the upper chamber and hCAF-BT cells in the lower chamber. After 24-hour synchronization with serum-free medium, cultures were switched to 1% FBS medium and maintained for 72 hours.

### 2.8 Establishment of stable sh-FAAH transfected cell lines

A lentiviral vector expressing shRNA targeting the FAAH gene (designed by GenePharma) and a negative control sh-NC were used. T24 cells were seeded at 5×10⁵ cells/well in 6-well plates, and transfection was performed using Lipofectamine 3000 when cell confluency reached 60%. Forty-eight hours post-transfection, puromycin (2μg/mL) was added for resistance screening, with stable knockdown cell lines obtained after 14 days of continuous culture.

### 2.9 CCK-8 assay for cell proliferation activity

T24 cells were adjusted to a single-cell suspension at 5×10³ cells/mL and seeded at 100μL/well in 96-well plates with 6 replicates per group. At 1, 2, 3, 4, 5 and 6 days post-seeding, 10μL CCK-8 reagent (Dojindo) was added, and after 2-hour incubation in the dark, absorbance at 450nm was measured using a microplate reader. Cell growth curves were plotted with time on the x-axis and OD values on the y-axis, with growth trends fitted using a four-parameter Logistic equation.

### 2.10 Statistical analysis

All data calculations and statistical analyses were performed in a 64-bit R (4.2 version) environment, with major R packages used described in the methods section. Data analysis workflows strictly adhered to FAIR principles. Statistical significance was denoted by * for P<0.05, ** for P<0.01, and *** for P<0.001. For multiple comparison scenarios (e.g., GSEA analysis or intergroup difference tests), Bonferroni correction was used by default.

## 3 Results

### 3.1 Taurine metabolic profiling and scoring in the BLCA microenvironment

**Figure 1:**
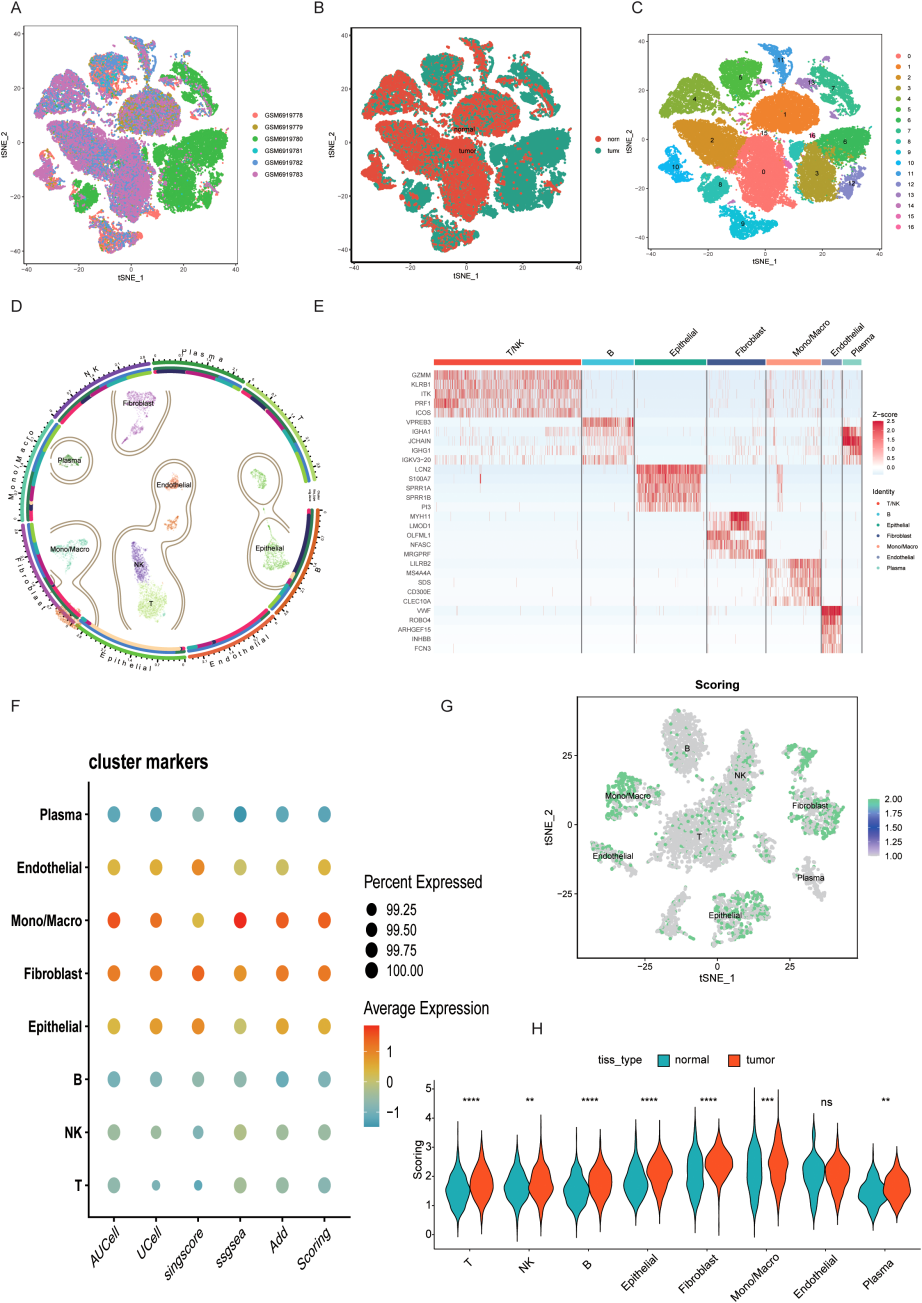
**(A-C)** UMAP plots display the clustering of different BLCA samples, including the distribution of cancer and adjacent tissues. **(D)** A circle plot of major cell dimensionality reduction clustering. **(E)** Expression of specific marker genes in different cell clusters. **(F, G)** Taurine characteristics of different cell clusters scored by multiple single-cell scoring tools. **(H)** Comparison of TMscore between BLCA samples and paired normal samples. ** P < 0.01, *** P < 0.001, **** P < 0.0001, ns: not significant.

To characterize the BLCA microenvironment, paired samples from six BLCA patients were subjected to rigorous quality control and dimensionality reduction, eliminating noise and redundant data (**Figures 1A, B**). The six samples mentioned above were further reduced in dimension to form 18 cell clusters. Subsequently, single-cell annotation techniques were employed to reveal that single-cell clustering had identified distinct cell populations, including mononuclear macrophages, plasma cells, epithelial cells, T/NK cells, B cells, endothelial cells, and fibroblasts (**Figures 1C, D**). The balanced distribution of these cell types in BLCA suggests a dynamic equilibrium critical for TME maintenance and progression. Marker gene analysis revealed cell-type-specific expression signatures (**Figure 1E**). Leveraging five single-cell scoring algorithms (UCell, AddModuleScore, AUCell, GSVA, singscore), we quantified taurine metabolic activity across cell types. Endothelial cells, macrophages, and epithelial cells exhibited the highest taurine metabolic scores (TMscore), as visualized in interactive bubble plots (**Figures 1F, G**). Comparative analysis between tumor and normal tissues highlighted differential TMscore in epithelial cells, fibroblasts, and macrophages, indicating tumor-specific reprogramming of taurine metabolism (**Figure 1H**).

### 3.2 Taurine-driven cell communication and differentiation trajectories in BLCA

**Figure 2:**
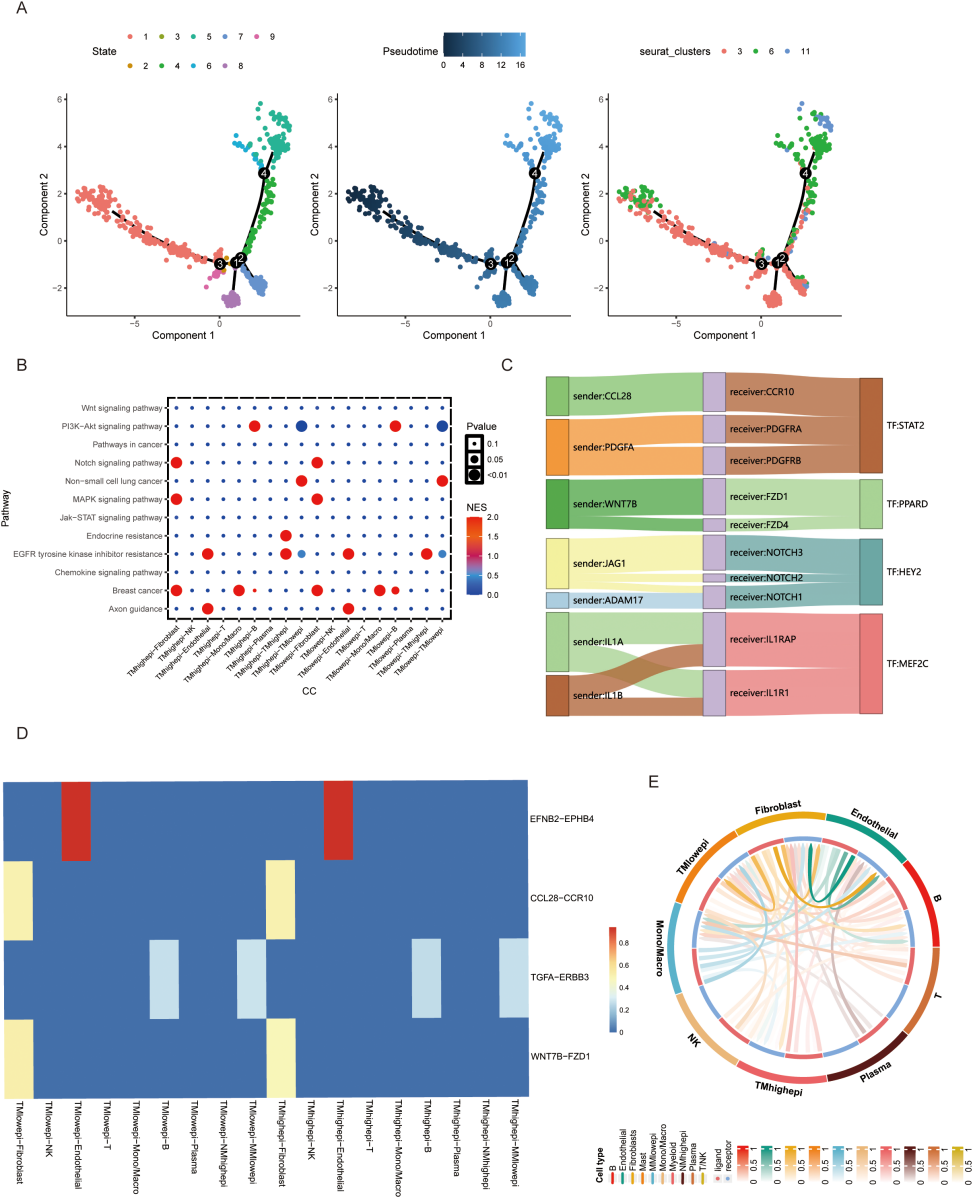
**(A)** Cell differentiation trajectory analysis of BLCA epithelial cell clusters. **(B)** Cell signaling of different cells in TMs low and TMs high groups. **(C, D)** Comparison of ligand-receptor relationships and corresponding transcription factors between TMs low and TMs high groups. **(E)** Interaction between epithelial cells in TMs low and TMs high groups and other cellular components in the microenvironment.

Using pseudotime analysis via Monocle 3, we reconstructed differentiation trajectories of TMscore-defined epithelial cell clusters. Eight distinct epithelial subpopulations were identified, with Cluster 6 displaying terminal differentiation markers at the trajectory endpoint (**Figure 2A**). Stratifying epithelial cells by TMscore median revealed enrichment of Notch signaling and EGFR tyrosine kinase inhibitor resistance pathways in high-TMscore cell**s (** **Figure 2B**). Transcription factor binding motif analysis identified HEY2 and PPARD as key regulators of intercellular crosstalk, with their binding sites enriched in differentially expressed gene promoters (**Figure 2C**). Ligand-receptor interaction modeling using CellChat uncovered enhanced communication between high-TMscore epithelial cells and fibroblasts/macrophages. The CCL28-CCR10 and WNT7B-FZD1 signaling axes emerged as dominant pathways mediating this crosstalk (**Figures 2D, E**), serving as molecular hubs for intercellular signaling.

### 3.3 Spatial heterogeneity of taurine metabolic dysregulation

**Figure 3:**
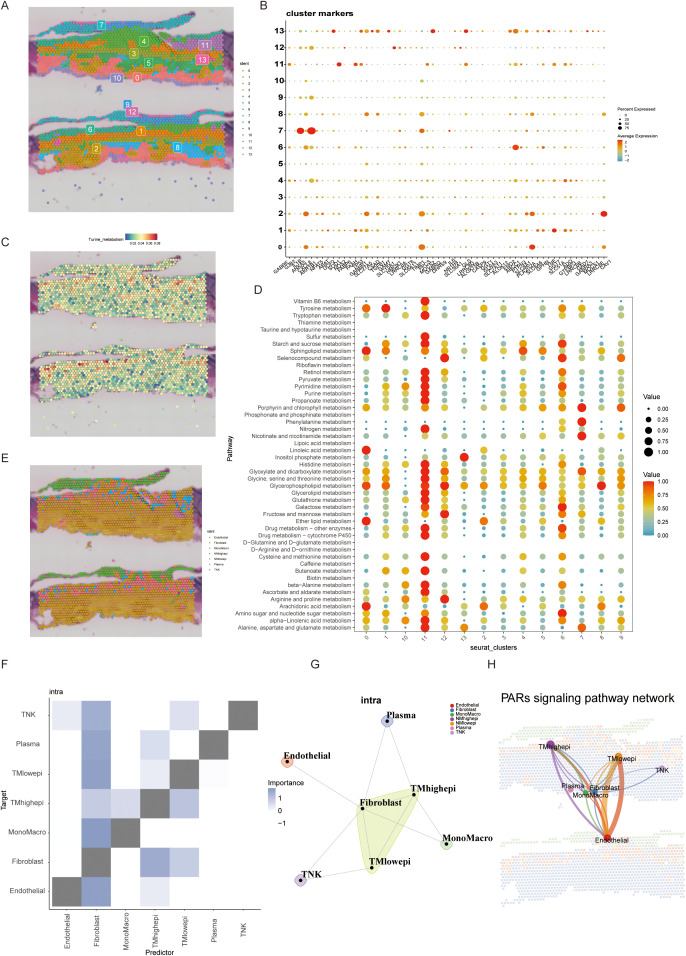
**(A, B)** Spatial transcriptome section annotation identifies 13 different BLCA cell spatial regions and corresponding regional markers. **(C)** Expression of the most significant genes in taurine metabolism across different spatial regions. **(D)** Activity of common tumor metabolic pathways in different spatial sections. **(E)** Cell annotation of spatial regions by integrating spatial transcriptome and single-cell data. **(F, G)** Cell subset interactions from a spatial perspective. **(H)** Spatial information of commonly activated pathways in taurine metabolism.

Integrating single-cell and spatial transcriptomics, we mapped 13 distinct cell subpopulations in BLCA tissue sections (**Figure 3A**). Spatial distribution of taurine metabolic genes (SLC27A5, CAV1) revealed pronounced heterogeneity, with tumor core regions exhibiting hyperactivation of taurine metabolism (**Figures 3B, C**). In the tumor microenvironment, metabolic crosstalk often has a systemic impact, where changes in one pathway can affect the entire network. To simultaneously analyze alterations in different metabolic pathways within the current spatial transcriptomics sections, we performed a metabolic pathway enrichment analysis via scMetabolism, which identified Cluster 11 as a metabolic hotspot (**Figure 3D**). RCTD-based spatial mapping showed high-TMscore epithelial cells preferentially localized to niches enriched in fibroblasts and macrophages (**Figures 3E–G**), validated by ligand-receptor co-expression and proximity ligation assays. PAR signaling pathway networks were identified as critical mediators of spatial cell–cell interactions in high-TMscore regions (**Figure 3H**), underscoring the role of metabolic reprogramming in shaping TME architecture.

### 3.4 Prognostic significance and genomic correlates of taurine metabolic dysregulation

**Figure 4:**
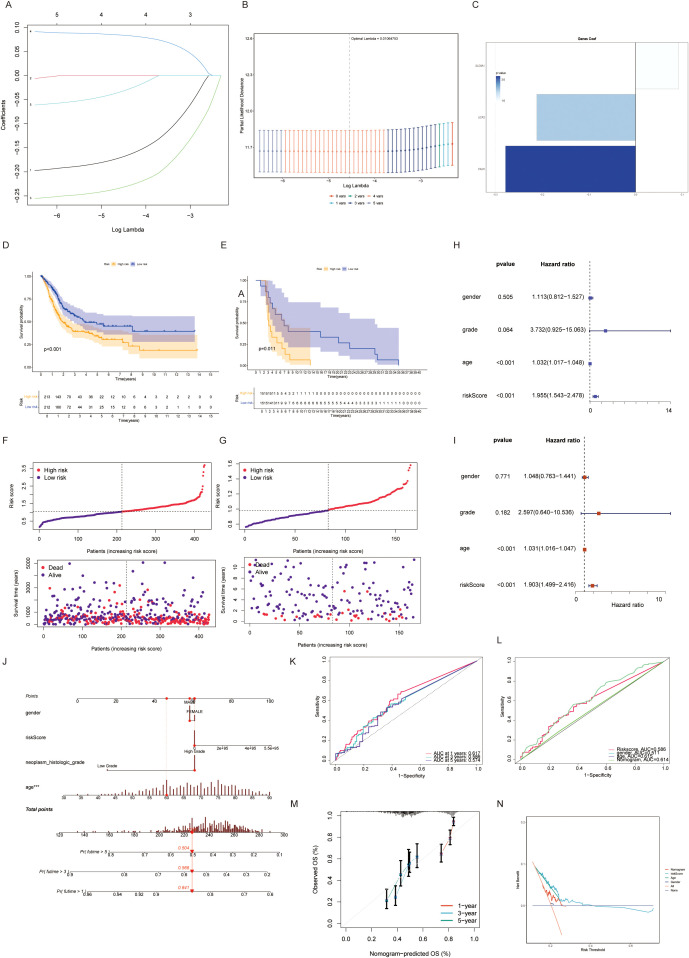
**(A, B)** Univariate COX regression combined with lasso regression analysis for model construction of taurine-related genes. **(C)** Importance assignment values of genes constructing the TMs criteria. **(D, E)** Discrimination and prognostic performance of TMs for BLCA patients in TCGA validation and training sets. **(F, G)** Risk plots for each patient in training and validation cohorts. **(H, I)** Evaluation of TMs criteria by univariate and multivariate cox risk scores. **(J-N)** Risk prediction constructed by dividing taurine metabolic disorder criteria.

**Figure 5:**
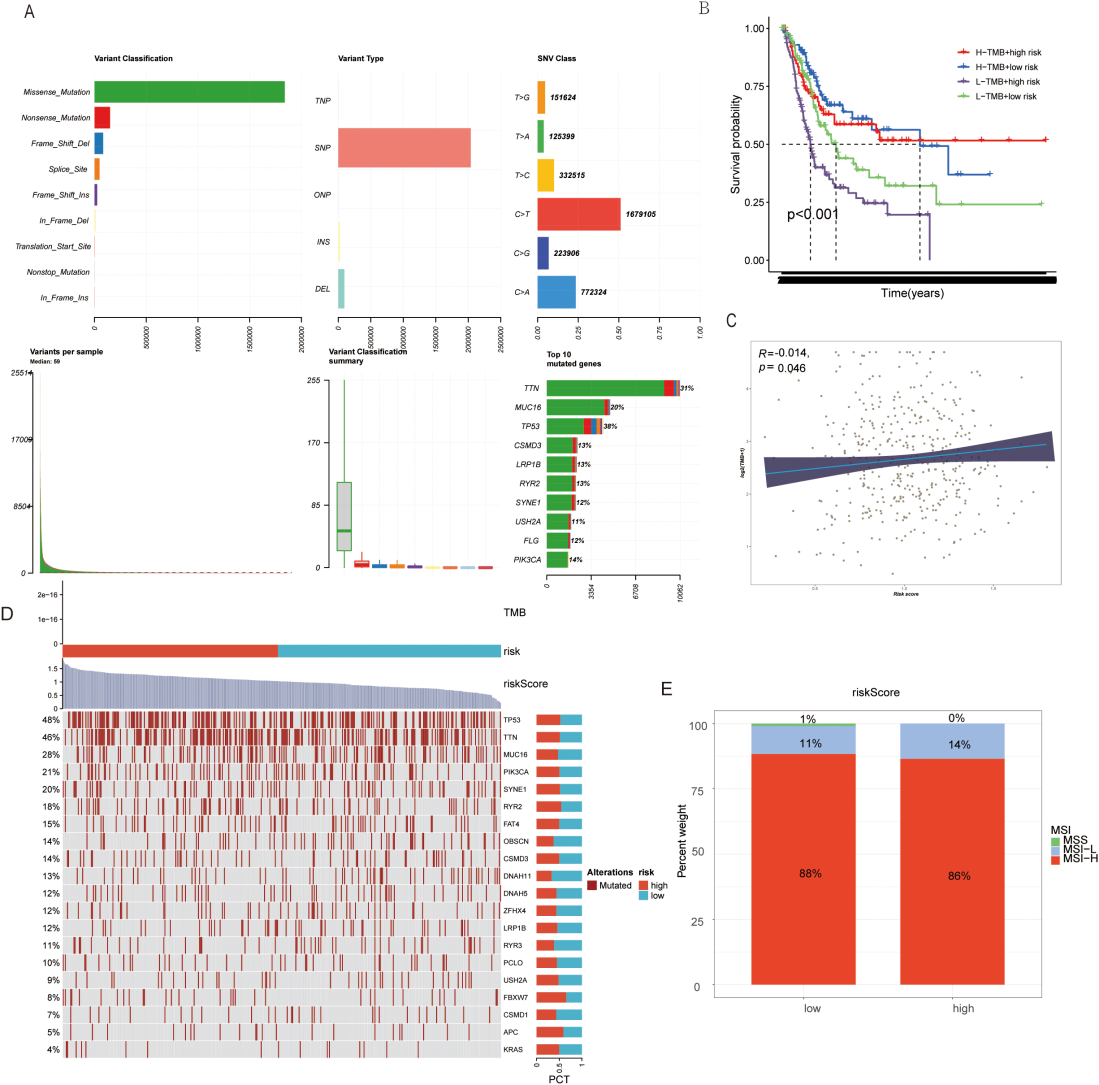
**(A)** Genomic mutation information changes in BLCA patients due to TMs criteria. **(B)** TMB mutation levels in TMs low and TMs high groups. **(C)** Correlation between TMs score and TMB in BLCA patient genomes. **(D)** Mutation status of common tumor-related genes in TMs low and TMs high groups. **(E)** MSI mutation status in TMs low and TMs high groups.

A taurine metabolic dysregulation index (TMs) was developed through integrative analysis of differentially expressed genes and survival data. Using Lasso-Cox regression, a multi-gene prognostic model was constructed, dividing patients into high- and low-TMs groups based on median scores (**Figures 4A–C**). Survival analysis revealed significantly shorter overall survival (OS) in high-TMs cohorts across TCGA-BLCA datasets (**Figures 4D, E**). Time-dependent ROC curves demonstrated superior prognostic accuracy of TMs compared to conventional clinicopathological features (AUC: 0.78 vs. 0.62, **Figures 4K, L**). Multivariate Cox analysis confirmed TMs as an independent prognostic factor (HR=1.87, P<0.001, **Figures 4H, I**). A clinical nomogram integrating TMs, age, and stage showed high calibration accuracy for 1-, 3-, and 5-year OS prediction (**Figures 4G, M, N**). Genomic profiling revealed high-TMs tumors harbored increased missense mutations, particularly in TP53, TTN, and RYR2 (**Figures 5A, D**). Combined TMs-TMB analysis identified a high-TMs/low-TMB subgroup with poorest prognosis (**Figures 5B, C**), suggesting synergistic effects of metabolic dysregulation and immune evasion. No significant association was observed between TMs and MSI, emphasizing TMB as the primary genomic correlate (**Figure 5E**).

### 3.5 Taurine metabolic dysregulation shapes the immune landscape of the BLCA tumor microenvironment

**Figure 6:**
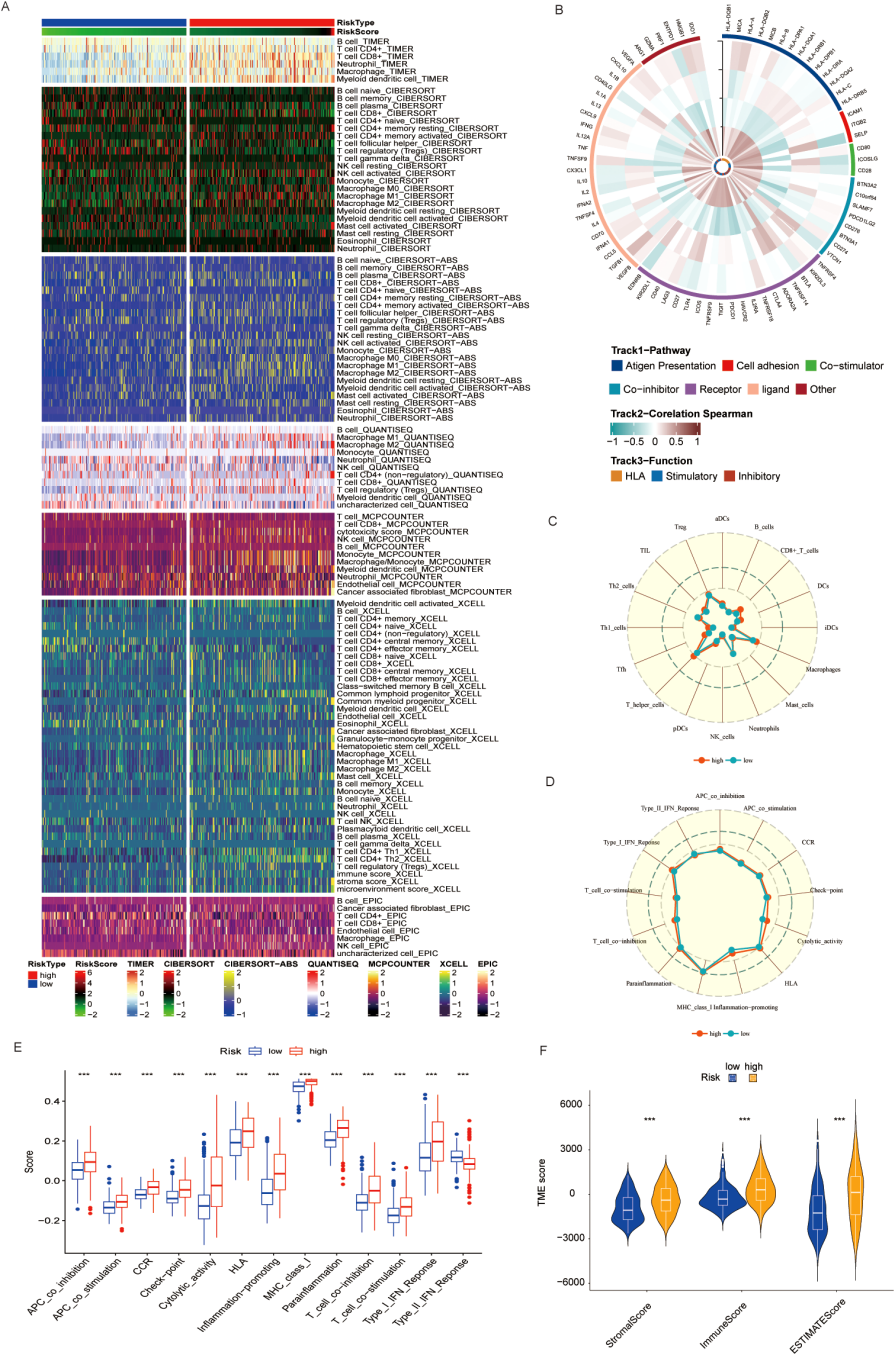
**(A)** Immune cell infiltration in populations divided by different TMs criteria using common TME evaluation methods. **(B)** Correlation of immune-related genes in different TMs groups. **(C, D)** Activation of immune-related pathways in TMs low and TMs high groups. **(E)** GSEA results also indicate immune functions in TMs low and TMs high groups. **(F)** Immune scores of TMs low and TMs high groups. *** P < 0.001.

Emerging evidence has highlighted the intricate crosstalk between metabolic reprogramming and the TME in BLCA. Building upon our previous findings implicating taurine metabolic dysregulation in shaping TME composition, we systematically profiled immune cell infiltration across TMs subgroups. Employing a suite of deconvolution algorithms, including CIBERSORT, TIMER, and xCELL, we observed that high-TMs tumors were characterized by marked enrichment of immune populations such as natural killer (NK) cells, memory B cells, CD8^+^ T cells, regulatory T cells (Tregs), and M2-polarized macrophages (**Figure 6A**). The coexistence of both immunostimulatory and immunosuppressive subsets suggests a complex immune reprogramming landscape accompanying taurine metabolic alterations. To further delineate the immunological shifts associated with TMs status, we analyzed the expression of immune-related genes, revealing heightened immune gene activity in the high-TMs group (**Figure 6B**). Single-sample gene set enrichment analysis (ssGSEA) corroborated these findings, showing significant enrichment of macrophage- and helper T cell-associated signatures in the high-TMs cohort (**Figures 6C, D**). In parallel, GSVA of canonical tumor immune pathways revealed increased activity in antigen presentation, immune checkpoint signaling, and cytotoxic effector functions in high-TMs tumors (**Figure 6E**), supporting a metabolically driven immunological remodeling. Quantitative assessment using the ESTIMATE algorithm further confirmed a consistent reduction in stromal, immune, and overall ESTIMATE scores in high-TMs tumors (**Figure 6F**), reflecting a shift toward an immunosuppressive and metabolically reprogrammed TME. Collectively, these data implicate taurine metabolism as a pivotal modulator of immune contexture in BLCA.

### 3.6 Taurine metabolic status predicts divergent immunotherapeutic responses in BLCA

**Figure 7:**
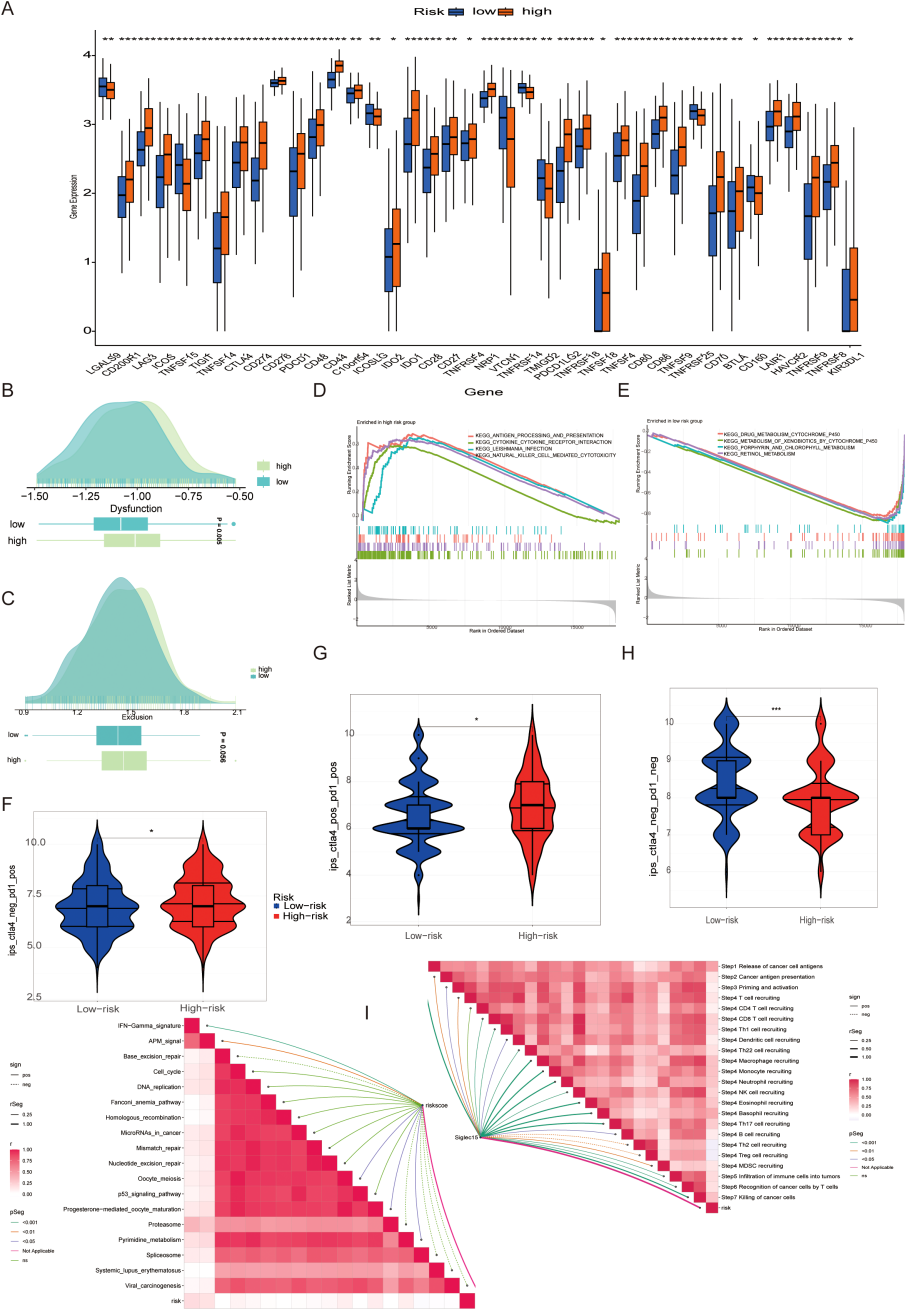
**(A)** Expression of immune checkpoint-related genes in different TMs groups. **(B, C)** Association between populations divided by different TMs criteria and immune exclusion and disorder. **(D, E)** Common functional enrichment in TMs low and TMs high groups. **(F-H)** Guiding value of TMs criteria for immunotherapy cohorts. **(I)** Correlation of immune response between PMs low and PMs high groups. * P < 0.05, ** P < 0.01, *** P < 0.001, **** P < 0.0001, ns: not significant.

Given the central role of the immune milieu in determining response to ICB, we next interrogated the impact of taurine metabolic imbalance on immunotherapy-related parameters. Expression profiling revealed that nearly all immune checkpoint genes were significantly upregulated in high-TMs tumors(**Figure 7A**), consistent with an immunosuppressive, potentially therapy-resistant state.To further quantify immune evasion and dysfunction, we employed the Tumor Immune Dysfunction and Exclusion (TIDE) framework. High-TMs tumors displayed elevated immune exclusion scores and reduced dysfunction scores (**Figures 7B, C**), suggesting active immune escape despite preserved effector function. GSVA analyses demonstrated concurrent upregulation of multiple immune response pathways, reinforcing the presence of a metabolically reinforced, yet immunologically active, tumor state (**Figures 7D, E**). Stratification by the Immuno-pheno-score (IPS)—a composite predictor of ICB response—revealed that TMs risk groups exhibited distinct sensitivities to PD-1 and CTLA-4 blockade, with notable differences in CTLA-4 responsiveness (**Figures 7F–H**). Correlation analyses confirmed that the TMs-derived risk score positively associated with multiple features of the cancer-immunity cycle, including tumor antigen release, presentation, and immune infiltration (**Figure 7I**). These findings underscore the potential of TMs status as a biomarker for predicting ICB efficacy and guiding therapeutic stratification in BLCA.

### 3.7 FAAH as a central mediator of taurine metabolic reprogramming and immune modulation

**Figure 8:**
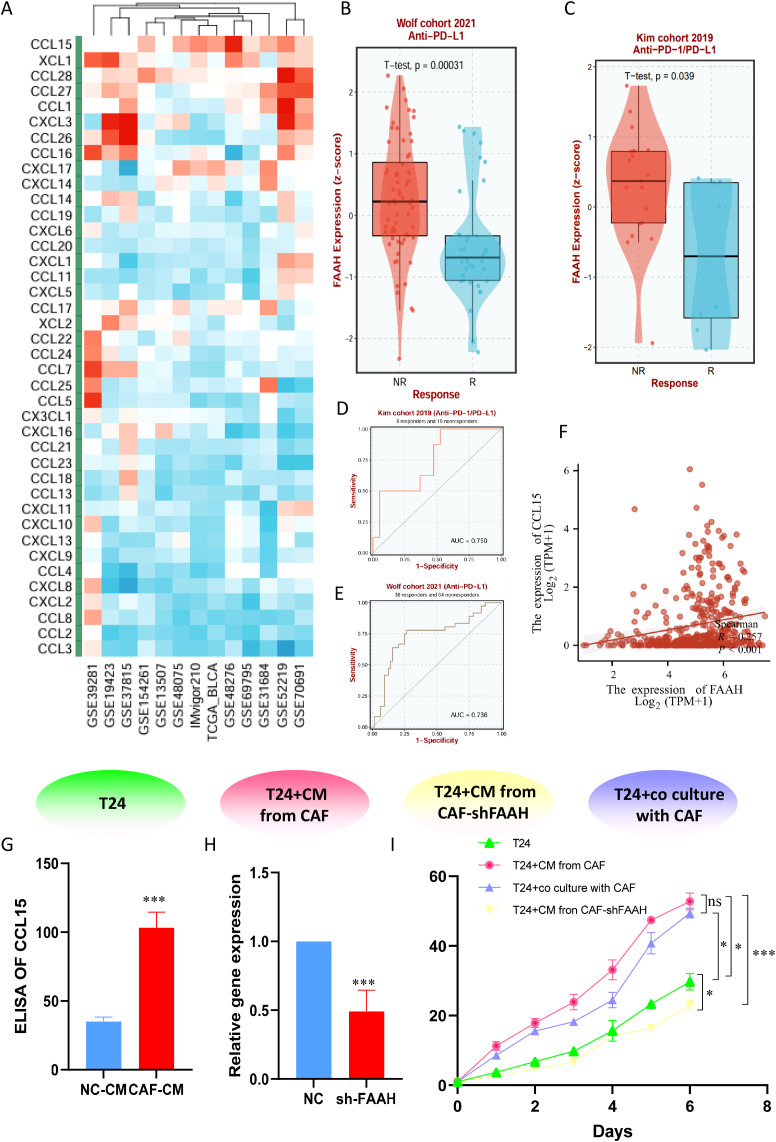
**(A)** Correlation between TMs criteria and cytokines in common BLCA patient cohorts. **(B, C)** Expression differences of FAAH between responding and non-responding patients in immunotherapy cohorts. **(D, E)** Therapeutic value of FAAH in different immunotherapy cohorts. **(F)** Expression correlation between FAAH and CCL15 in TCGA dataset. **(G)** Expression level of CCL15 in CAF supernatant. **(H)** Stable transfection to knockdown FAAH expression in immortalized CAFs. **(I)** CCK-8 assay comparing the viability of bladder cancer cells in co-culture systems. * P < 0.05, *** P < 0.001, ns: not significant.

Considering the value of TMs mentioned above for tumor immunity, we attempted to address this potential relationship through internal mechanisms. We constructed an ssGSEA score using taurine disorder criteria and evaluated the relationship between this criterion and common cytokines in the BLCA population. Cross-cohort correlation analyses demonstrated a robust association between TMs and the chemokine CCL15 (**Figure 8A**), a fibroblast-and tumor-derived factor implicated in immune recruitment and suppression. Given its high loading score in the taurine metabolic signature, FAAH emerged as a candidate effector of metabolic reprogramming in BLCA. These results suggest that FAAH may mediate immune modulation via paracrine signaling. In immunotherapy-treated cohorts, high FAAH expression was significantly associated with non-responder status (NR), and showed predictive value across clinical datasets (**Figures 8B–E**). The scatter plot reveals the correlation between FAAH and CCL15 in TCGA data (**Figure 8F**). Notably, conditioned media (CM) derived from CAFs exhibited significantly higher levels of CCL15 compared to that from normal fibroblasts (**Figure 8G**), implicating a FAAH-CCL15 axis in fibroblast-mediated immune modulation. To mechanistically validate these findings, we established a series of co-culture systems: control, T24 + CAF CM, T24 + CAF CM with FAAH knockdown (CAF-sh FAAH), and direct T24-CAF co-culture. CCK-8 demonstrated that CAF-derived CM enhanced T24 proliferation, an effect abrogated by FAAH knockdown (**Figures 8H, I**). Together, these data position FAAH as a functional driver of taurine metabolic reprogramming and an actionable target for reversing fibroblast-mediated immunosuppression in BLCA.

## 4 Discussion

Metabolic reprogramming is a central strategy enabling tumor cells to withstand microenvironmental stress, with its interface with the immune system emerging as a pivotal axis in precision oncology (21–24). Tumor cells are recognized to construct an immunosuppressive microenvironment by remodeling metabolic pathways such as glucose and amino acids. For example, glutamine deprivation inhibits T-cell activation, while enhanced fatty acid oxidation promotes regulatory T-cell differentiation. Also, abnormal taurine metabolism recruits macrophages via CCL15, weakening immune surveillance. Targeting metabolic nodes like IDO and CD73, or combining with immune checkpoint inhibitors, can reverse metabolic immune evasion. In-depth analysis of the metabolism-immunity interaction mechanism is expected to provide new strategies for developing efficient combination therapies and predicting treatment responses, driving the precision development of tumor immunotherapy. In bladder cancer (BLCA), aberrant taurine metabolism orchestrates a profound remodeling of the immune landscape, giving rise to an inflammation-driven oncogenic ecosystem and unveiling novel avenues for tumor–immune crosstalk (21, 25). While metabolic rewiring confers a survival advantage to tumor cells, it concurrently redefines immune cell states (26, 27). Taurine, a key derivative of sulfur-containing amino acid metabolism, displays marked cell-type-specific dysregulation within the BLCA microenvironment. Endothelial cells, macrophages, and epithelial cells exhibit elevated taurine metabolic activity (TMscore), which may perturb immune surveillance through multiple mechanisms. First, taurine deficiency, by reducing antioxidant capacity, exacerbates oxidative stress and mitochondrial dysfunction in CD8^+^ T cells, culminating in exhaustion. Second, tumor cells with hyperactive taurine metabolism outcompete immune cells for taurine uptake via transporters such as SLC6A6, inducing endoplasmic reticulum stress and upregulation of inhibitory receptors including PD-1 (17, 28). Taurine depletion has also been shown to impair dendritic cell (DC) maturation through mTORC1 inhibition, consistent with the suppressed antigen presentation signature observed in high-TMs tumors (28).

The immune-microenvironment of is characterized by diverse immune cell infiltration, yet tumor cells establish complex immune escape networks through metabolic reprogramming (24, 27, 29). Using single-cell sequencing, this study clustered eight core cell populations. Notably, high-TMs epithelial cells formed strong interactions with fibroblasts and macrophages via ligand-receptor pairs such as CCL28-CCR10 and WNT7B-FZD1. Such cell communication may promote secretion of pro-cancer inflammatory cytokines (e.g., IL-6, TGF-β) while inhibiting effector T cell recruitment. Clinical prognostic analysis revealed that the taurine metabolic dysregulation index (TMs) not only independently predicted overall survival (OS) but also outperformed traditional pathological features in predictive efficacy, suggesting taurine metabolic status may serve as a novel biomarker for BLCA patient stratification. Additionally, spatial transcriptomic analysis identified heterogeneous overexpression of core taurine metabolic genes (e.g., SLC27A5, CAV1) in tumor core regions, which were significantly co-localized with fibroblasts and macrophages. This metabolic-structural synergy may further exacerbate immune suppression through mechanisms such as local microenvironmental acidification and nutrient deprivation (30). Single-cell trajectory analysis showed that high-TMs epithelial cells exhibited enriched abnormal activation of the Notch signaling pathway, potentially promoting stemness maintenance and immune escape. Ligand-receptor interaction networks revealed significant enrichment of the CCL28-CCR10 axis in high-TMs groups, which recruits CCR10+ Tregs to tumor nests, forming local immunosuppressive microenvironments. Spatial metabolic analysis further demonstrated that taurine metabolic abnormalities in high-TMs regions were accompanied by enhanced glutamine catabolism, whose metabolite lactate inhibits CD8+ T cell cytotoxic granule release by reducing microenvironmental pH. This multi-dimensional cell interaction network not only reshapes immune cell functional states but also provides multiple potential intervention nodes for targeting the metabolism-immunity axis (24).

High-TMs tumors are marked by three hallmarks: immunological dysregulation, genomic instability, and spatially heterogeneous metabolic activity. Despite increased infiltration of effector cells such as NK cells and memory B cells, the high-TMs subgroup exhibited elevated Tregs and M2-like macrophages, alongside heightened expression of immune checkpoints including PD-L1 and CTLA-4, indicative of a functionally immunosuppressive state (30, 31).Mechanistically, taurine may directly modulate immune cell function: its excess promotes Treg differentiation via the AHR pathway and suppresses glycolysis in CD8^+^ T cells, impairing cytotoxic function. Genomically, high-TMs tumors harbored higher missense mutation burdens, particularly in TP53 and TTN. Notably, high-TMs patients with low tumor mutational burden (TMB) exhibited significantly poorer OS, implicating taurine dysregulation in the destabilization of genomic integrity—potentially through oxidative stress–induced DNA repair defects—which may blunt the immunogenicity typically associated with high TMB. Although immune checkpoint blockade (ICB) demonstrates efficacy in BLCA, therapeutic response is heavily modulated by the metabolic milieu (24, 32).Elevated immune exclusion in high-TMs tumors suggests a physical immune barrier, potentially formed via fibroblast recruitment through the WNT7B–FZD1 axis. While increased checkpoint expression in high-TMs tumors implies therapeutic susceptibility, concurrent antigen-presenting cell (APC) activation and cytotoxic signaling argue for metabolic co-targeting to overcome resistance. For example, SLC6A6 inhibition may restore taurine availability in T cells and potentiate ICB efficacy (15, 33).Intriguingly, TMs and TMB interact: high-TMB/high-TMs patients displayed relatively favorable outcomes, while low-TMB/high-TMs tumors fared worst—likely due to taurine-induced downregulation of MHC-I, diminishing the antigen-presentation advantage conferred by high TMB. Microsatellite instability (MSI), however, remained unaltered across TMs strata, indicating that taurine metabolism modulates immunogenicity independently of DNA mismatch repair, offering novel therapeutic possibilities for MSI-H patients.

The genes screened in this study, such as FAAH, UCP2, and SLC8A1, form the core of the TMs model. As a fatty acid amide hydrolase, high expression of FAAH is associated with the accumulation of the endocannabinoid 2-AG, which inhibits T cell migration by activating CB2 receptors. UCP2, a mitochondrial uncoupling protein, exhibits abnormal expression that reduces mitochondrial membrane potential and ATP production in T cells, thereby suppressing effector functions. Abnormal sodium/calcium exchange mediated by SLC8A1 may induce immune tolerance by disrupting the calcium signaling threshold for T cell activation. The abnormal expression of these genes not only intersects with taurine metabolic pathways but also directly affects the energy metabolism and signal transduction of immune cells, suggesting that interventions targeting these molecules may simultaneously regulate metabolic dysregulation and immunosuppression. Our experiments in this study preliminarily revealed that FAAH affects tumor-stroma interactions by regulating CCL15 secretion, but the effect of immune cell recruitment needs to be further validated.

In summary, this study revealed the central regulatory role of taurine metabolic dysregulation in the BLCA immune-microenvironment through single-cell transcriptomics, spatial omics, and survival analysis. Abnormal taurine metabolism not only induces immunosuppression by reshaping the epithelial-stromal cell interaction network but also synergizes with genomic instability and spatial metabolic heterogeneity to drive tumor progression. However, the causal relationship between taurine metabolic abnormalities and BLCA development requires validation in gene-edited animal models, and the specific regulatory pathways of genes such as FAAH and UCP2 in the metabolism-immunity axis need further clarification. Future research could focus on developing small-molecule drugs targeting taurine transporters or improving T cell function through taurine supplements, combined with spatial metabolomics to monitor dynamic microenvironmental changes in real time, thereby promoting a paradigm shift in bladder cancer therapy from single-target approaches to metabolism-immunity network regulation.

## Data Availability

The original contributions presented in the study are included in the article. Further inquiries can be directed to the corresponding author.

## References

[1] Li D Wang Z Yu Q Wang J Wu R Tuo Z. Tracing the evolution of sex hormones and receptor-mediated immune microenvironmental differences in prostate and bladder cancers: from embryonic development to disease. Adv Sci (Weinh). (2025) 12:e2407715. doi: 10.1002/advs.202407715 40007149 PMC11967776

[2] Li R Hensley PJ Gupta S Al-Ahmadie H Babjuk M Black PC. Bladder-sparing therapy for bacillus calmette-guérin-unresponsive non-muscle-invasive bladder cancer: international bladder cancer group recommendations for optimal sequencing and patient selection. Eur Urol. (2024) 86:516–27. doi: 10.1016/j.eururo.2024.08.001 39183090

[3] Liatsos GD Mariolis I Hadziyannis E Bamias A Vassilopoulos D. Review of BCG immunotherapy for bladder cancer. Clin Microbiol Rev. (2025) 38:e0019423. doi: 10.1128/cmr.00194-23 39932308 PMC11905372

[4] Zabeti Touchaei A Vahidi S. Unraveling the interplay of CD8 + T cells and microRNA signaling in cancer: implications for immune dysfunction and therapeutic approaches. J Transl Med. (2024) 22:1131. doi: 10.1186/s12967-024-05963-5 39707465 PMC11662517

[5] Qin J Li Z Su L Wen X Tang X Huang M. Expression of transferrin receptor/TFRC protein in bladder cancer cell T24 and its role in inducing iron death in bladder cancer. Int J Biol Macromol. (2024) 274:133323. doi: 10.1016/j.ijbiomac.2024.133323 38908617

[6] Yu P Zhu C You X Gu W Wang X Wang Y. The combination of immune checkpoint inhibitors and antibody-drug conjugates in the treatment of urogenital tumors: a review insights from phase 2 and 3 studies. Cell Death Dis. (2024) 15:433. doi: 10.1038/s41419-024-06837-w 38898003 PMC11186852

[7] Huang P Wang J Yu Z Lu J Sun Z Chen Z. Redefining bladder cancer treatment: innovations in overcoming drug resistance and immune evasion. Front Immunol. (2025) 16:1537808. doi: 10.3389/fimmu.2025.1537808 39911393 PMC11794230

[8] Liu Q Guan Y Li S. Programmed death receptor (PD-)1/PD-ligand (L)1 in urological cancers: the “all-around warrior” in immunotherapy. Mol Cancer. (2024) 23:183. doi: 10.1186/s12943-024-02095-8 39223527 PMC11367915

[9] Li Z Li J Bai X Huang X Wang Q. Tumor microenvironment as a complex milieu driving cancer progression: a mini review. Clin Transl Oncol. (2025) 27:1943–52. doi: 10.1007/s12094-024-03697-w PMC12033186 39342061

[10] Noeraparast M Krajina K Pichler R Niedersüß-Beke D Shariat SF Grünwald V. FGFR3 alterations in bladder cancer: Sensitivity and resistance to targeted therapies. Cancer Commun (Lond). (2024) 44:1189–208. doi: 10.1002/cac2.v44.10 PMC11483561 39161208

[11] Singh A Raja D Kaushal S Seth A Singh P Sharma A. Phenotypic characterization of tumor associated macrophages and circulating monocytes in patients with Urothelial carcinoma of bladder. Immunol Res. (2025) 73:66. doi: 10.1007/s12026-025-09624-7 40195201

[12] van Dorp J van der Heijden MS. The bladder cancer immune micro-environment in the context of response to immune checkpoint inhibition. Front Immunol. (2023) 14:1235884. doi: 10.3389/fimmu.2023.1235884 37727793 PMC10505825

[13] Stary D Bajda M. Taurine and creatine transporters as potential drug targets in cancer therapy. Int J Mol Sci. (2023) 24:3788. doi: 10.3390/ijms24043788 36835201 PMC9964810

[14] Santulli G Kansakar U Varzideh F Mone P Jankauskas SS Lombardi A. Functional role of taurine in aging and cardiovascular health: an updated overview. Nutrients. (2023) 15:4236. doi: 10.3390/nu15194236 37836520 PMC10574552

[15] Flemming A. Tumour cell consumption of taurine exhausts CD8(+) T cells. Nat Rev Immunol. (2024) 24:306. doi: 10.1038/s41577-024-01032-6 38632489

[16] Baliou S Adamaki M Ioannou P Pappa A Panayiotidis MI Spandidos DA. Protective role of taurine against oxidative stress (Review). Mol Med Rep. (2021) 24:605. doi: 10.3892/mmr.2021.12242 34184084 PMC8240184

[17] Seneff S Kyriakopoulos AM. Taurine prevents mitochondrial dysfunction and protects mitochondria from reactive oxygen species and deuterium toxicity. Amino Acids. (2025) 57:6. doi: 10.1007/s00726-024-03440-3 39789296 PMC11717795

[18] Jiang Y Tao Q Qiao X Yang Y Peng C Han M. Targeting amino acid metabolism to inhibit gastric cancer progression and promote anti-tumor immunity: a review. Front Immunol. (2025) 16:1508730. doi: 10.3389/fimmu.2025.1508730 40018041 PMC11864927

[19] Weinstein JN Collisson EA Mills GB Shaw KR Ozenberger BA Ellrott K. The Cancer Genome Atlas Pan-Cancer analysis project. Nat Genet. (2013) 45:1113–20. doi: 10.1038/ng.2764 PMC3919969 24071849

[20] Li T Fu J Zeng Z Cohen D Li J Chen Q. TIMER2.0 for analysis of tumor-infiltrating immune cells. Nucleic Acids Res. (2020) 48:W509–w14. doi: 10.1093/nar/gkaa407 PMC7319575 32442275

[21] Zhang M Wu J Zhang Y Shang H. Recent advances of neoadjuvant immunotherapy for urothelial bladder cancer. Ann Surg Oncol. (2024) 31:5851–9. doi: 10.1245/s10434-024-15725-8 38995447

[22] Wang Q Huang X Zhou S Ding Y Wang H Jiang W. IL1RN and PRRX1 as a prognostic biomarker correlated with immune infiltrates in colorectal cancer: evidence from bioinformatic analysis. Int J Genomics. (2022) 2022:2723264. doi: 10.1155/2022/2723264 36483329 PMC9726255

[23] Zhao S Wang Q Liu Y Zhang P Ji W Xie J. Interaction, immune infiltration characteristics and prognostic modeling of efferocytosis-related subtypes in glioblastoma. BMC Med Genomics. (2023) 16:248. doi: 10.1186/s12920-023-01688-4 37853449 PMC10583324

[24] Yang L Wang Q He L Sun X. The critical role of tumor microbiome in cancer immunotherapy. Cancer Biol Ther. (2024) 25:2301801. doi: 10.1080/15384047.2024.2301801 38241173 PMC10802201

[25] Scholtes MP de Jong FC Zuiverloon TCM Theodorescu D. Role of bladder cancer metabolic reprogramming in the effectiveness of immunotherapy. Cancers (Basel). (2021) 13:288. doi: 10.3390/cancers13020288 33466735 PMC7830378

[26] Peng M Chu X Peng Y Li D Zhang Z Wang W. Targeted therapies in bladder cancer: signaling pathways, applications, and challenges. MedComm (2020). (2023) 4:e455. doi: 10.1002/mco2.455 38107059 PMC10724512

[27] Zhang B Yang L He Y Han D Qi P Shang P. Role and mechanisms of noncoding RNAs in the regulation of metabolic reprogramming in bladder cancer (Review). Int J Mol Med. (2023) 52:79. doi: 10.3892/ijmm.2023.5282 37477143

[28] Roh J Im M Chae Y Kang J Kim W. The involvement of long non-coding RNAs in glutamine-metabolic reprogramming and therapeutic resistance in cancer. Int J Mol Sci. (2022) 23:14808. doi: 10.3390/ijms232314808 36499136 PMC9738059

[29] Wan R Pan L Wang Q Shen G Guo R Qin Y. Decoding gastric cancer: machine learning insights into the significance of COMMDs family in immunotherapy and diagnosis. J Cancer. (2024) 15:3580–95. doi: 10.7150/jca.94360 PMC11134438 38817875

[30] van Puffelen JH Keating ST Oosterwijk E van der Heijden AG Netea MG Joosten LAB. Trained immunity as a molecular mechanism for BCG immunotherapy in bladder cancer. Nat Rev Urol. (2020) 17:513–25. doi: 10.1038/s41585-020-0346-4 32678343

[31] Jian N Yu L Ma L Zheng B Huang W. BCG therapy in bladder cancer and its tumor microenvironment interactions. Clin Microbiol Rev. (2025) 38:e0021224. doi: 10.1128/cmr.00212-24 40111053 PMC12180517

[32] Woolbright BL Ayres M Taylor JA 3rd. Metabolic changes in bladder cancer. Urol Oncol. (2018) 36:327–37. doi: 10.1016/j.urolonc.2018.04.010 29773495

[33] Cao T Zhang W Wang Q Wang C Ma W Zhang C. Cancer SLC6A6-mediated taurine uptake transactivates immune checkpoint genes and induces exhaustion in CD8(+) T cells. Cell. (2024) 187:2288–304.e27. doi: 10.1016/j.cell.2024.03.011 38565142

